# Analysis of Flow Cytometry Data by Matrix Relevance Learning Vector Quantization

**DOI:** 10.1371/journal.pone.0059401

**Published:** 2013-03-18

**Authors:** Michael Biehl, Kerstin Bunte, Petra Schneider

**Affiliations:** 1 Johann Bernoulli Institute for Mathematics and Computer Science, University of Groningen, Groningen, The Netherlands; 2 CITEC, Faculty of Technology, University of Bielefeld, Bielefeld, Germany; 3 CEDAM, School of Clinical and Experimental Medicine, University of Birmingham, Birmingham, United Kingdom; Mount Sinai School of Medicine, United States of America

## Abstract

Flow cytometry is a widely used technique for the analysis of cell populations in the study and diagnosis of human diseases. It yields large amounts of high-dimensional data, the analysis of which would clearly benefit from efficient computational approaches aiming at automated diagnosis and decision support. This article presents our analysis of flow cytometry data in the framework of the DREAM6/FlowCAP2 Molecular Classification of Acute Myeloid Leukemia (AML) Challenge, 2011. In the challenge, example data was provided for a set of 179 subjects, comprising healthy donors and 23 cases of AML. The participants were asked to provide predictions with respect to the condition of 180 patients in a test set. We extracted feature vectors from the data in terms of single marker statistics, including characteristic moments, median and interquartile range of the observed values. Subsequently, we applied Generalized Matrix Relevance Learning Vector Quantization (GMLVQ), a machine learning technique which extends standard LVQ by an adaptive distance measure. Our method achieved the best possible performance with respect to the diagnoses of test set patients. The extraction of features from the flow cytometry data is outlined in detail, the machine learning approach is discussed and classification results are presented. In addition, we illustrate how GMLVQ can provide deeper insight into the problem by allowing to infer the relevance of specific markers and features for the diagnosis.

## Introduction

We present in this article our main results obtained in the context of the DREAM6/FlowCAP2 *Molecular Classification of Acute Myeloid Leukemia Challenge 2011* [1]–[3]. This challenge was organized in a joint effort by the *Dialogue for Reverse Engineering Assessments and Methods* (DREAM) project [3]–[6] and the *Flow Cytometry: Critical Assessment of Population Identification Methods* (FlowCAP) initiative [2].

Flow cytometry constitutes a powerful technique which is widely used in medical research and clinical practice for the study and diagnosis of various diseases [7]. Flow cytometry measurements typically yield a quantitative description of several tens or even hundreds of thousands of cells in a given sample. Light scatter and fluorescence properties are used to identify deviations from normal cell size or structure and to quantify functional properties in terms of, e.g., protein marker expressions [7], [8]. The amount of available data, its high dimension, and the complexity of the diagnosis tasks trigger a significant interest in systems for automated analysis and decision support.

Along these lines, the DREAM6/FlowCAP2 challenge addressed the analysis of given flow cytometry data, representing peripheral blood and bone marrow samples of, in total, 359 subjects. Some of these corresponded to cases of Acute Myeloid Leukemia (AML) and the ultimate goal was to predict the condition of a number of patients whose diagnosis was unknown to the participants. Hence, the goal of the challenge could be formulated as a machine learning problem: From the given example data with known diagnoses, criteria were to be inferred which then allowed for the classification of the *test samples*.

We extracted feature vectors from the data in terms of a few characteristic quantities, summarizing the statistics of the observed marker values. Predictions were obtained by means of a specific machine learning technique termed Generalized Matrix Relevance Learning Vector Quantization (GMLVQ) [9]–[11]. This prototype based method extends standard Learning Vector Quantization [12], [13] by using *Adaptive Distance Measures in Relevance LVQ*, which motivated the acronym and team name *Admire-LVQ*. In the challenge, our team achieved the best possible performance with respect to the required test set prediction.

In the following section a description of the data set and our analysis is given. Thereafter we present and discuss our main results and the obtained prediction. We conclude with a brief outlook on possible extensions and future work.

## Data Set and Analysis

In this section we first describe the extraction of features from the given data. The specific machine learning analysis based on Generalized Matrix Relevance Learning Vector Quantization is outlined. Furthermore, its validation in terms of the given data set is discussed.

The data set provided in the challenge comprised 359 subjects. For each of these, a varying number of cells, on the order of a few thousands, had been analysed by means of flow cytometry, see [2], [3] for details. The first 179 subjects served as the training data; label information 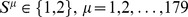 was provided, specifying 23 subjects as AML patients 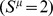. The remaining 156 subjects are referred to as *healthy donors* 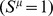 throughout this contribution. Note that the latter group of subjects includes a number of patients with a diagnose different from AML [14].

The task was to predict the diagnosis with respect to a test set of 180 subjects for which no label information was provided. The total number of AML cases in the test set, 20, was also disclosed to the participants. However, this information was not exploited in our approach. We have analysed the *transformed* and *compensated* flow cytometry data as provided by the organizers of the challenge [2], [3]. In our analysis we omitted the non-specific isotope control data representing non-human binding antibodies, which corresponds to *tube 8* in the data set [3].

In clinical practice, a possible workflow is to sort cells according to a small number of *gating* variables in a first step, identifying potentially degenerate or immature cells. Subsequently, the selected cells are analysed according to the remaining markers, aiming at a reliable diagnosis and potential identification of the AML subtype [7], [8]. In our approach we follow a simpler, more direct strategy in which we omit cell specific information. After visual inspection in terms of histograms we decided to represent the data by a limited number of statistical characteristics per patient and marker. Moreover, we took into account all markers at once in order to assign each subject to one of the two classes in a single processing step.

### Feature Extraction and Normalization

A key step in the design of a classifier in this challenge was the extraction of appropriate features from the provided data. The data corresponding to *tubes 1–7* represents 31 characteristic quantities per cell: the so-called *Forward Scatter* on linear scale (FS Lin), the *Sideward Scatter* on logarithmic scale (SS Log), and 29 fluorescence intensities on logarithmic scale quantifying the expression of various surface proteins. All of these quantities are referred to as *markers* in the following. Table 1 lists the considered markers and the index 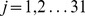 which we refer to in the analysis.

**Table 1:** List of the 31 markers used in the analysis.

1	2	3	4	5	6	7	8
FS Lin	SS Log	CD45- EDC	IgG1- FITC	Kappa- FIT	CD7- FITC	CD15- FITC	CD14- FITC
9	10	11	12	13	14	15	16
HLADR- FITC	CD5- FITC	IgG1- PE	Lambda- PE	CD4- PE	CD13- PE	CD11c- PE	CD117- PE
17	18	19	20	21	22	23	24
CD19- PE	IgG1- PC5	CD19- PC5	CD8- PC5	CD16- PC5	CD64- PC5	CD34- PC5	CD3- PC5
25	26	27	28	29	30	31	
IgG1- PC7	CD20- PC7	CD2- PC7	CD56- PC7	CD33- PC7	CD38- PC7	CD10- PC7	

Note that the potential *gating* markers FS Lin, SS Log, and CD45-EDC were provided for all cells in the data set. The other 28 markers were measured in one tube only, representing a sub-population of cells per subject. We rescaled all markers by the respective largest possible value as to limit all observations to the interval 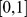.

FS Lin can be interpreted as a measure of cell size, while SS Log roughly quantifies intracellular granularity [7]. Note furthermore that the expression of IgG1 was measured by means of four different binding antigens. In our analysis, however, the corresponding values were treated as four independent markers (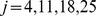), formally.

**Figure 1:**
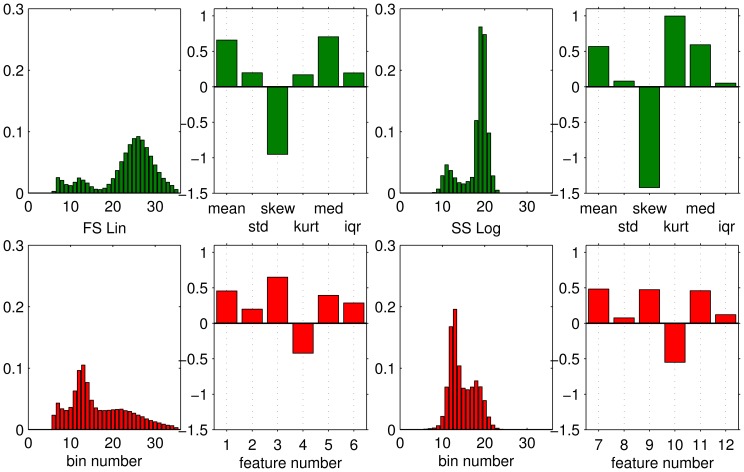
Example histograms and extracted features: FS Lin and SS Log. Histograms and extracted features correspond to one healthy donor (subject 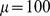, upper panels) and one AML patient (subject 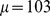, lower panels), respectively. Histograms display the frequency of a particular marker value for visual inspection. Six features are extracted per patient and marker, corresponding to mean, standard deviation, skewness, kurtosis, median, and interquartile range of the observed frequency of marker values, cf. Eq. (1). Here the first 12 components of the 186-dim. feature vectors are displayed before z-score transformation.

**Figure 2:**
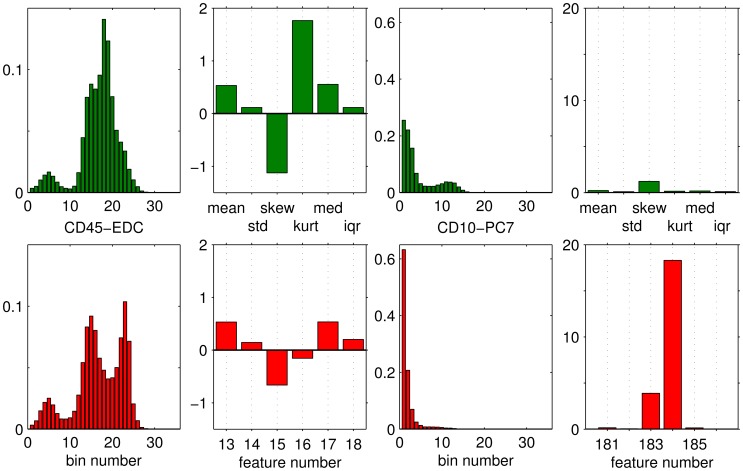
Example histograms and extracted features: CD45-EDC and CD10-PC7. For further description see Figure 1. The quantities displayed here correspond to features 13–18 and 181–186 of the 186-dim. vectors before z-score transformation.

For the purpose of a first, visual inspection, we computed histograms corresponding to the frequency of marker values in the training set. Figures 1 and 2 display histograms of 4 example markers: FS Lin 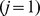, SS Log 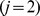, CD45-EDC 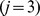, and CD10-PC7 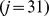 for one patient per class (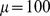 and103). The main purpose of Figures 1 and 2 is to illustrate the extraction of feature vectors from the sample data which is described in the following.

For each patient and marker a varying number 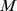 of cell measurements, typically a few thousands, were made available. In our analysis, we did not make use of cell specific information, as it is done frequently in terms of a so-called *gating* procedure in clinical practice [7], [8]. We extracted information only on the level of single marker statistics over the entire population of cells. A direct classification of histograms using, for instance, entropic distance measures or statistical divergences would be feasible here [15], [16]. We resorted, however, to reducing the information to only six quantities per marker which summarize the characteristics of the corresponding histogram. We denote by 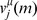 the value measured for marker 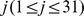 in individual cell 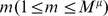 of patient 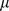. From the available data we determined the following quantities:
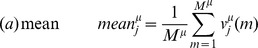


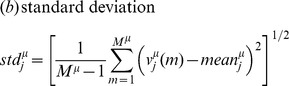


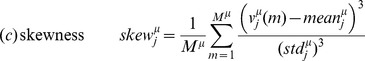


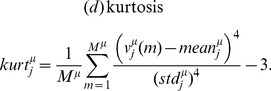
(1)


In addition we computed (e) median 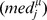 and (f) interquartile range 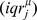 in the set of observed values 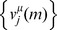. The skewness is a measure of the asymmetry, with positive values indicating that more weight is contained in the *left side of the histogram*. The kurtosis quantifies how sharply peaked a histogram is. Note that in the above defined 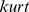, sometimes termed *excess kurtosis* in the literature, a constant 3 is subtracted yielding 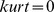 in case of normal densities.

Hence we obtained, for each patient 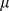, a set of 6 quantities per marker. A particular subject was subsequently represented by the concatenated vector 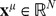 of 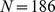 characteristic features. As one example, the skewness of marker 17 (CD19-PE, see Table 1) observed for patient 42 corresponds to component 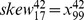 of the feature vector 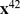 since 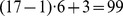.

The features representing markers 1–3 (FS Lin, SS Log, CD45-EDC) and marker 31 (CD10-PC7) are shown for one example subject from each class in Figures 1 and 2, together with the corresponding histograms.

In the training processes described in the following, we applied an additional z-score transformation: Given a (sub-)set of 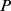 training examples we computed for 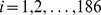 the quantities
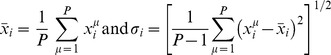
and rescaled all features in training, validation or test data by subtracting the mean 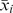 and subsequently dividing by 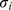. Consequently, the transformed features display zero mean and unit variance in the actual training set. While the transformation did not affect the classification performance, it enhances the interpretability of the results, in particular with respect to the relevance matrix, see below.

### Matrix Relevance Learning Vector Quantization

We employed Generalized Matrix Relevance Learning Vector Quantization (GMLVQ) for the analysis of the obtained feature vectors. This highly flexible and powerful variant of LVQ is described in detail in [9]–[11]. Here we employed the algorithm in its simplest setting with one prototype per class and a single, global relevance matrix as defined below.

The two classes, i.e. healthy donors (class 1) and AML patients (class 2), are represented by the prototype vectors 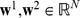, respectively. Given a particular z-score-transformed feature vector 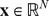 representing one of the patients, its distance from the prototypes is determined as
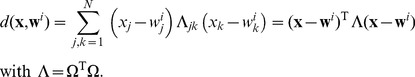
(2)


Here 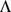 and 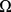 are 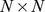 matrices and the specific parameterization of the distance guarantees non-negativity of the measure:
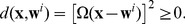
(3)


In a simple *Nearest Prototype Classification* (NPC) scheme, a feature vector 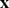 is assigned to class 1 if 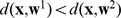 and to class 2, else. While 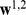 serve as typical representatives of the classes, elements 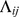 of the symmetric matrix 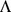 can be interpreted as to quantify the relevance of a pair of feature dimensions 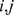 in the classification scheme.

Both, prototypes and relevances, are determined in the same supervised training process. Given a set of 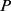 examples 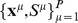 with class labels 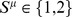, training is guided by the minimization of the cost function [9], [17], [18]

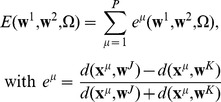
(4)where the index 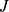 corresponds to the *correct* prototype with 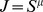 while 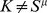 identifies the *wrong* prototype. In general, the objective of training can be further specified by introducing a function 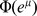 in the cost function, e.g. a *sigmoidal* [17]. Here, we resorted to the simple case 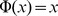. Note that the contribution 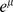 of a single example to the cost function satisfies 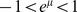. It is negative if 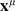 is classified correctly and its absolute value relates to the *margin* of the classification.

Alternatively we refer to the closely related *score* 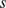 which is computed as
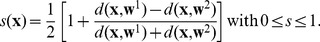
(5)


A value 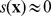 indicates that feature vector 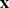 is assigned to class 1, healthy donors, with high certainty. Large values close to 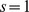 signal confident classification as an AML patient (class 2). The NPC scheme can be reformulated as assigning vector 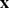 to class 1 if 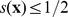 and to class 2 else. While the score may serve as a relative measure of certainty in GMLVQ, it should not be interpreted directly as a probability for AML. Note that any monotonically increasing function 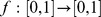 could be used to transform 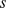 without modifying the actual ordering of patients according to 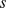.

We implemented the iterative optimization of 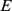, cf. Eq. (4), by means of a gradient descent procedure with respect to the adaptive quantities 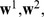 and 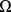. At iteration step 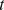, updates along the normalized gradients and subsequent normalization of 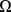 were performed:
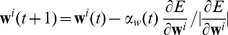


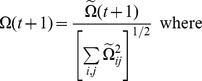


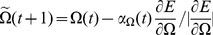
(6)with the time-dependent step sizes 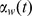 and 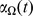. The full form of the gradient terms is given in [9], [11], [19], for instance. We employed gradient descent with *waypoint averaging and step size control*, which has been introduced and described in greater detail in [19]: After a gradient step, Eq. (6), the achieved value of the cost function 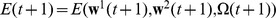 is compared with 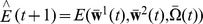 where



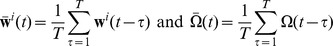
corresponds to the position in search space, on average over the last 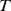 updates. The observation of 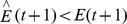 signals oscillatory behavior of the iteration. In this case, we set 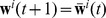 and 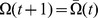 and reduced the step sizes by a factor 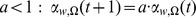. All results presented here were obtained with parameters 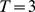 and 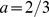 in the waypoint averaging scheme. Initial step sizes were 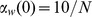 for prototypes and 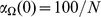 for matrix updates, respectively. In the problem at hand, the obtained classification scheme and error rates turned out very robust with respect to the choice of these parameters.

For a given training set, we initialized prototypes 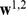 close to the corresponding class conditional means with small random deviations; similarly we chose the initial 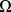 close to the 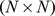 identity matrix:
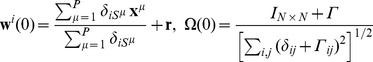
(7)where the Kronecker–Delta 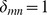 if 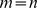 and 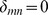 if 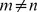. The components of 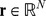 and all elements of 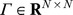 were generated independently according to a uniform density 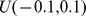. Results were found to depend only very weakly on details of the initialization.

### Validation

In order to evaluate the performance of the GMLVQ classifier before applying it to the test set, we employed a validation scheme based on randomized subsets of the available training data. In every run we selected ca. 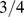 of the data from each class randomly, i.e. 17 of the 23 AML examples and 117 of the 156 healthy donors. These 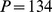 example data were used for training the GMLVQ system while the remaining 45 served as a validation set. The random split of the data was repeated 50 times and, if not stated otherwise, results presented in this section were obtained on average over the validation runs.

**Figure 3:**
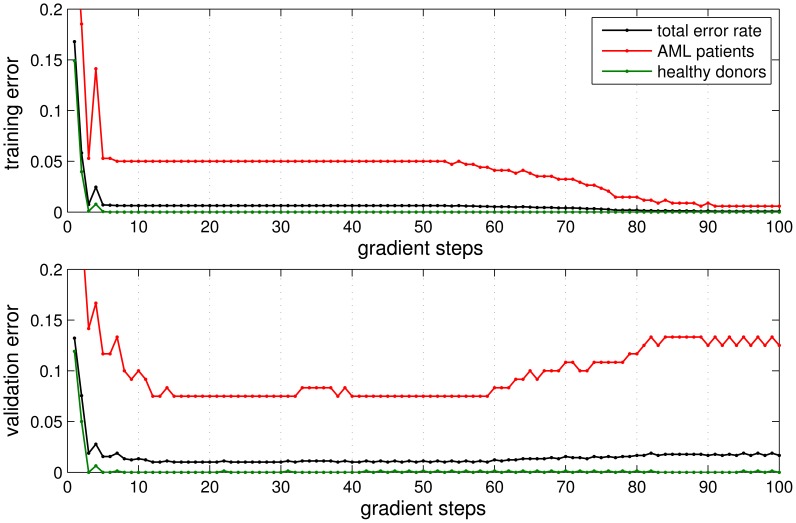
Learning curves in the validation procedure. Class specific and total error rates of Nearest Prototype Classification, corresponding to 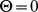 in Eq. (8), on average over 50 randomized validation runs. The upper panel corresponds to the performance in the respective training set, the lower panel displays error rates with respect to the validation set. The curves correspond to including patient 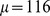 in training or validation set.

Figure 3 displays the averaged error rates of naïve Nearest Prototype Classification in the course of gradient based training. Note that an over-fitting effect was observed: Performing more than ca. 60 training steps decreased the error rates with respect to training examples to very low values. At the same time, however, validation set performance deteriorated. Closer inspection revealed that this effect was essentially due to patient 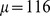, listed as a case of AML in the training set. If contained in the validation set, this patient was consistently misclassified by the NPC scheme. On the contrary - if employed for training - the system achieved agreement with the label, eventually, but at the expense of an increased error rate in the validation set.

Based on this observation, we employed an *early stopping* strategy, terminating the training process after 40 gradient steps. When omitting patient 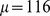 from the training set or re-labeling the subject as healthy donor, the learning curves converged smoothly and overfitting was not observed anymore. Moreover, we obtained virtually the same classification, i.e. the same order of scores with respect to the test set patients in all these scenarios. The precise numerical results reported in the following section were obtained by means of the early stopping strategy including subject 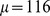 labelled as an AML case 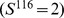.

**Figure 4:**
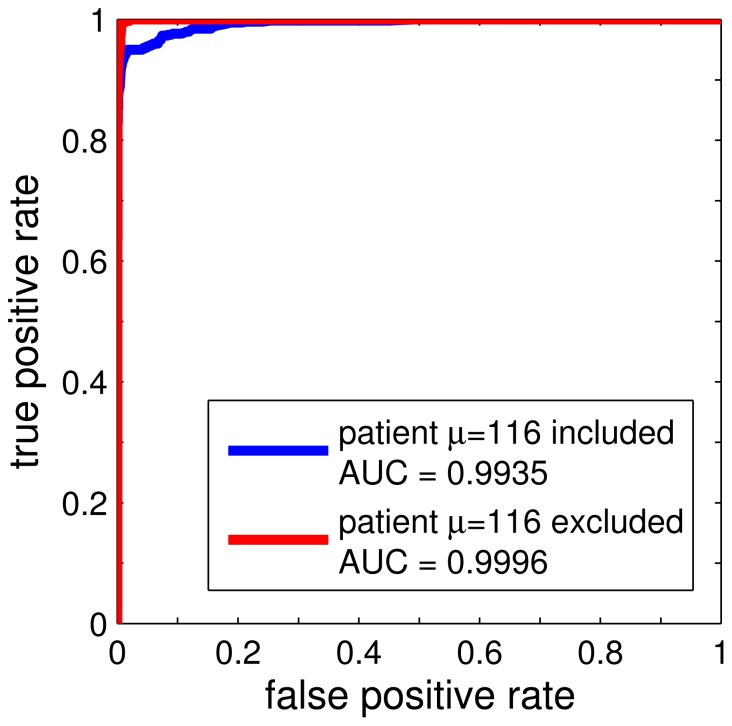
Validation set performance. Threshold-averaged ROC as obtained in the validation runs using labeled data. The curves correspond to using the data set including patient 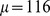 (lower, blue line) and excluding patient 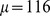 from the analysis completely (upper, red line), respectively.

In addition to the error rates of the naïve NPC scheme we also evaluated the validation set performance in terms of the Receiver Operating Characteristics (ROC) [20]. By introducing a threshold 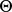, the GMLVQ scheme can be biased with respect to one of the two classes:
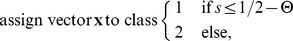
(8)with the score 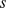 defined in Eq. (5) For thresholds in the range 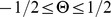 we computed the corresponding class-wise error rates with respect to the validation set on average over the 50 training runs, yielding the threshold-averaged ROC curves [20] displayed in Figure 4.

The ROC analysis revealed very high sensitivity (true positive rate) and specificity (1 - false positive rate) with respect to the validation set performance, the corresponding Area Under Curve being 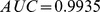 [20]. In addition, removal of patient 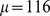 from the data set resulted in an almost perfect ROC with 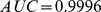. Given the close to error–free classification we refrained from employing complementary performance measures such as precision/recall or other characteristics [20]. For the same reason, we did not compare the validation performance of the simple GMLVQ scheme with more sophisticated settings or alternative classifiers.

## Results and Discussion

Final results, including the test set scores, were obtained using all 179 training samples for training. In addition, we performed an average over 50 randomized intializations in order to rule out an influence of the initial configuration of the GMLVQ system. In each run, 40 gradient steps were performed with waypoint averaging and step size control as described above. The final test set scores were obtained on average over the 50 randomized training runs.

Before discussing the outcome of the GMLVQ training in terms of prototypes and relevances we present the actual test set predictions.

### Test Set Prediction

**Figure 5:**
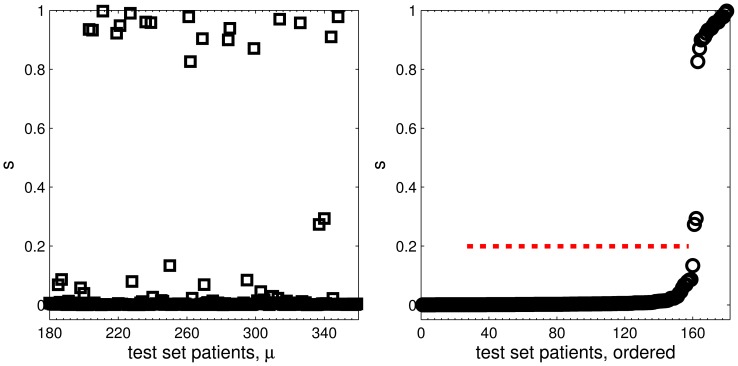
Test set predictions. GMLVQ based score 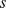 vs. patient number 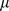 in the test set (left panel) and ordered according to 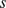 (right panel). The dotted line marks an example posterior choice of the threshold 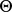, cf. Eq. (5), for crisp classification yielding correct prediction of 20 AML patients in the test set.

Figure 5 displays the GMLVQ based scores 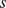, cf. Eq. 5, with respect to the 180 test set patients. Values close to 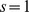 correspond to patients that are identified as AML patients with high certainty, while small 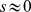 correspond to a classification as healthy donor. It would be very interesting to study potential correlations of the scores 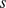 with additional information about the patients, e.g. measures of the *severity* of the AML cases. Unfortunately such information was not disclosed and is not available for the given data set.

Although it was known to the participants that the test set contained 20 AML cases, we did not make explicit use of this information. In the GMLVQ training, a threshold value does not have to be specified. The result in terms of scores 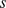 and the corresponding ranking of test set patients is independent of the actual number of AML cases. In a practical context, and if a *crisp* classification is the goal, the actual value of 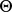 should be set according to domain expert (user) preferences concerning the compromise between sensitivity and specificity. The example threshold value marked in the right panel of Figure 5 was chosen a posteriori for illustration purposes only and is neither a result nor a parameter of the training process. With respect to performance in the challenge it is irrelevant.

The comparison with the unknown test set labels after submission of the predictions [3] revealed that the 20 patients with highest GMLVQ score 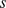 corresponded precisely to the 20 AML patients in the test set. Hence, we achieved the best possible prediction according to Receiver Operator Characteristics or other evaluation methods like Precision/Recall, which only depend on the order of scores and the corresponding ranking of patients.

**Figure 6:**
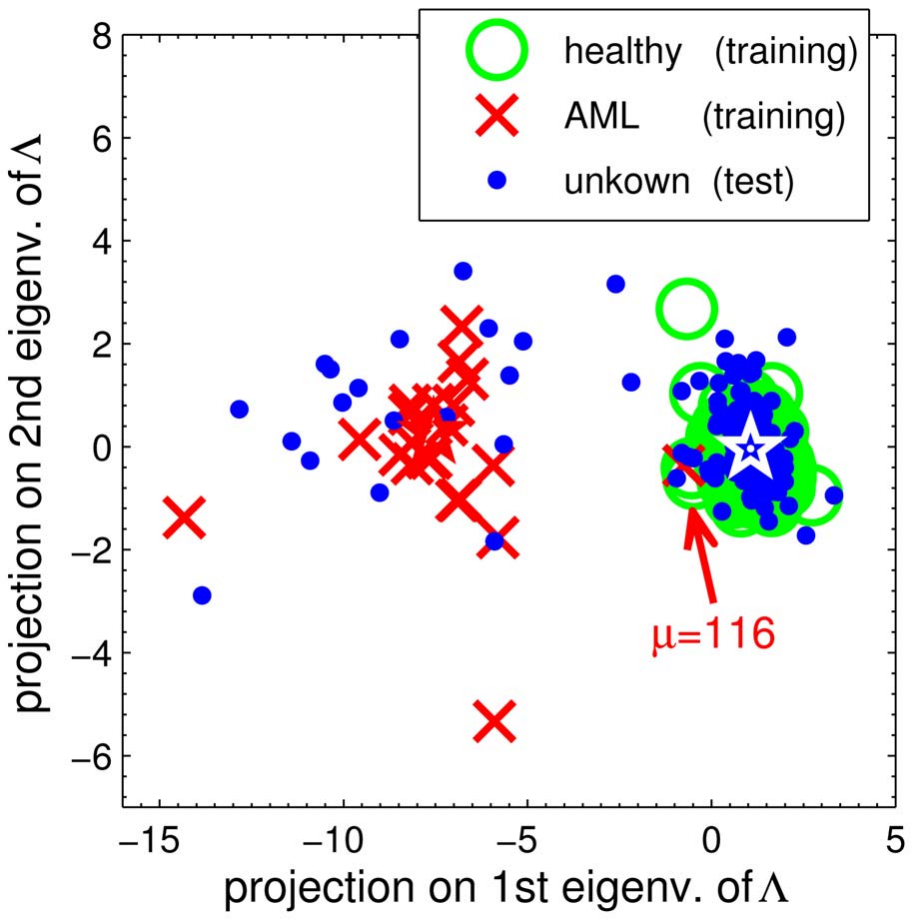
Visualization of the data set as obtained by GMLVQ. Projections of normalized feature vectors on the leading eigenvectors of 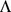 are displayed. Green circles correspond to healthy donors, red symbols mark AML patients in the training set, while blue dots represent test set data. Stars indicate the positions of the prototypes. The red arrow marks patient 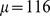 in the training set, who is labeled as AML but is misclassified persistently for a large range of thresholds 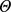, cf. Eq. (5).

The obtained classifier can be illustrated in terms of a two-dimensional visualization: Figure 6 displays the training and test data in terms of projections on the leading eigenvectors of the relevance matrix 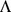 [21]. Two rather well separated clusters can be identified which reflect the assignment of classes. Note that the training set subject (patient 116) that was consistently misclassified by the NPC scheme is, indeed, located in the cluster representing healthy donors. This relates to the overfitting behavior discussed in greater detail in the previous section.

It is remarkable that error-free classification of the test set data was obtained by a number of teams who extracted different features from the data and used a variety of classification approaches [1]. For example, Vilar et *al.* employed a histogram based classifier in connection with the Kullback-Leibler divergence used as an entropic distance measure [16]. Amar et *al.* also extracted statistical moments from the data, but applied Support Vector Machine Regression, subsequently [22]. Logistic Regression was applied successfully by Manninen et *al.* [23]. Strickert and Seifert based their predictions on a method termed Correlative Matrix Mapping [24]. Using their software library *Jstacs* [25], Keilwagen and Grau built a weighted ensemble of classifiers which also achieved perfect classification.

An additional ranking of the best performing teams was suggested by the organizers in retrospect [1], [3], [26]. It hinges on interpreting the submitted scores as probabilistic assignments and on the reliability of the test set labels. In our opinion, the suggested posterior ranking according to, e.g., the Pearson correlation between scores 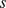 and the test set class labels is questionable, see also the DREAM6 discussion forum at [3].

### Characteristics of the GMLVQ Classifier

Apart from yielding the actual classification scheme, the GMLVQ analysis provides insights into the structure of the data which become available by inspection of the prototypes and relevance matrix. The interpretability of the classifier has proven useful in several applications and facilitates discussions with the respective domain experts [27], [28].

**Figure 7:**
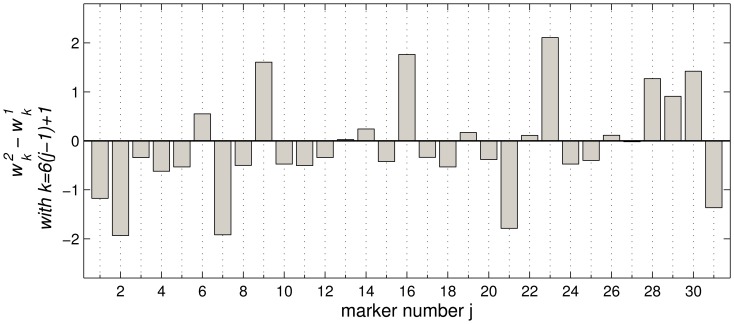
GMVLQ prototypes. Components of the difference vector 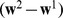 corresponding to the feature 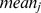, cf. Eq. (1), as represented by the AML prototype 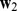 and healthy donor prototype 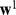. Positive bars indicate that 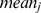 is typically greater in AML patients than in healthy donors.

Figure 7 visualizes the difference vector 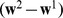 of prototypes representing healthy donors (1) and AML patients (2), respectively. For the sake of clarity, we have shown only the 31 components which correspond to the features 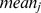, cf. Eq. (1 a). A positive difference corresponds to markers which display a greater value in the AML prototype compared to the typical healthy donor in the data set, examples being HLA-DR-FITC 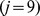, CD117-PE 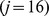, and CD34-PC5 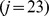. Example markers which display reduced values 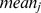 in AML patients are CD15-FITC 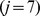, CD16-PC5 (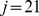), and CD10-PC7 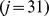.

In addition we analysed the resulting relevance matrix 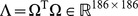. We focused on the diagonal elements 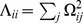 which formally accumulate the importance of feature 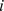 for the resulting classification.

The direct interpretation of 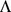 is simplified if all features assume values on the same order of magnitude. This condition was realized here by the explicit z-score transformation mentioned above. Moreover, it is important to note that, given a particular set of feature vectors 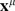 and prototypes 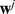, a continuum of matrices 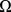 may exist which yield the same distances 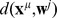, cf. Eq. (3) and, hence, the same classification scheme with respect to the training data. This ambiguity problem is particularly pronounced for inter-dependent or highly correlated features in high dimension. Resulting difficulties concerning the interpretation of 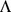 in terms of feature relevances are discussed in depth in [28]. There, schemes are suggested for posterior regularization which provide unique, interpretable 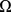 and 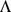 which we also applied here: Note that arbitrary vectors from the null-space or kernel 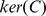 of the matrix.
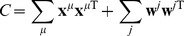
can be added to the rows of a given 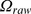 without changing the GMVLQ cost function (4) and the actual classification of training data. In [28] a column space projection is suggested in order to remove contributions from 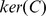 [28]:
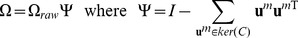
(9)is constructed from the eigenvectors 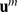 of 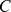 with eigenvalues zero.

Zero eigenvalues of 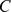 reflect the presence of linear dependent or strongly correlated features and the corresponding eigenvectors mark directions in input space in which training samples and prototypes do not vary. In the data considered here, one clearly expects dependencies between related markers, the four versions of IgG1 being an obvious example. In addition, extracted features like 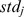 and 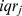 or 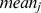 and 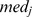 should be strongly correlated.

For the following discussion we determined 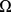 by means of a posterior column space projection (9) with 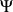 retaining only the leading eigendirections of 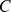 with eigenvalues 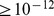. Thereafter, the matrix was normalized again to satisfy 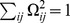 and we computed the regularized 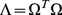.

It is remarkable that, in the given problem, this posterior regularization has very little influence on the test set classification. In particular, the ordering of test set scores obtained with the regularized system is the same as the one presented in the previous section. This suggests that the correlations and dependencies observed in the training set are already representative for the entire data. In [28] example problems are presented where the posterior regularization has a non-trivial effect also on test set performance.

**Figure 8:**
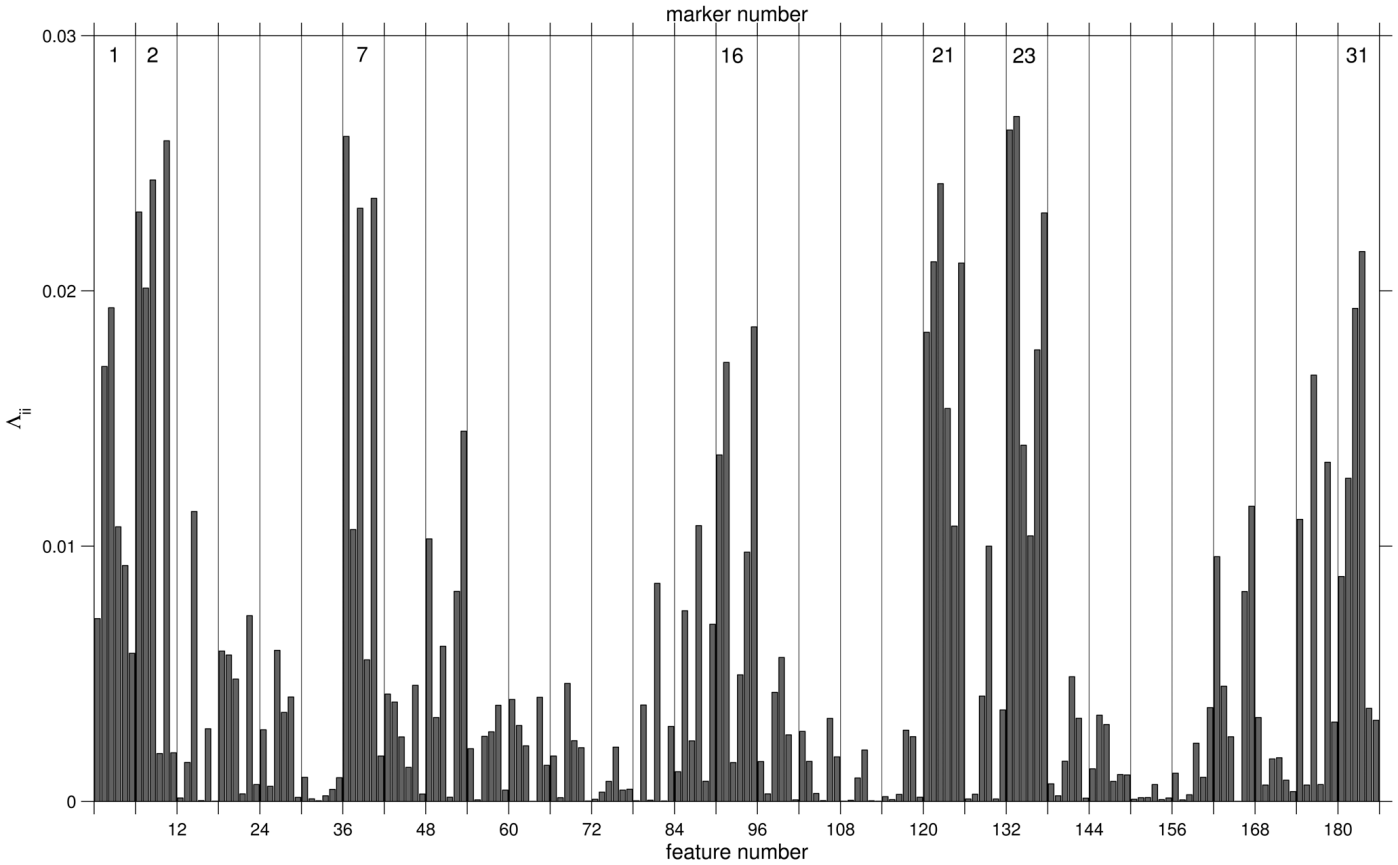
Relevance profile. Diagonal relevances 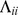 of features 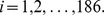 Vertical grid lines separate the groups of 6 quantities corresponding to each of the 31 markers, cf. Table 1. Marker numbers are given explicitly for 7 highly relevant ones.

Figure 8 displays the diagonal entries of 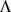 for all 186 features. After regularization, the heuristic interpretation of 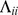 as the relevance or significance of feature 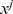 in the classification is justified [28]. The figure displays the features in groups of six, corresponding to the 31 markers, cf. Table 1.

A relatively small number of markers appears to contribute the most significant features: FS-Lin (1), SS-Log (2), CD15-FITC (7), CD117-PE (16), CD16-PC5 (21), CD34-PC5 (23), and CD10-PC7 (31). A more detailed discussion of the obtained 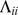 provides further, valuable information: For instance, the histogram shape as measured by skewness and kurtosis appears to be of minor importance with respect to marker 16 (CD117-PE), while measures of the corresponding histogram width (std, iqr) seem to represent significant differences between AML patients and healthy donors. On the contrary, for CD10-PC7 (marker 31) skewness and kurtosis carry most discriminative power.

While several of the above mentioned markers have been discussed as relevant in the context of AML in the literature, see e.g. [7], [28], [29], [30], their expression characteristics can vary a lot with the actual AML variant. For instance, both, HLA-DR positive and HLA-DR negative types of AML exist [31], the same applies to several other markers.

Due to the limited size of the data set and because information about AML subtypes was not disclosed, one should not over-interpret the results presented above. It is very likely that our findings in terms of relevances and prototypes are highly specific for the provided data set which seems to represent particular types of AML only. Nevertheless, our results demonstrate the interpretability of the GMLVQ approach and illustrate how the method could be used for efficient biomarker selection in collaboration with domain experts.

**Figure 9:**
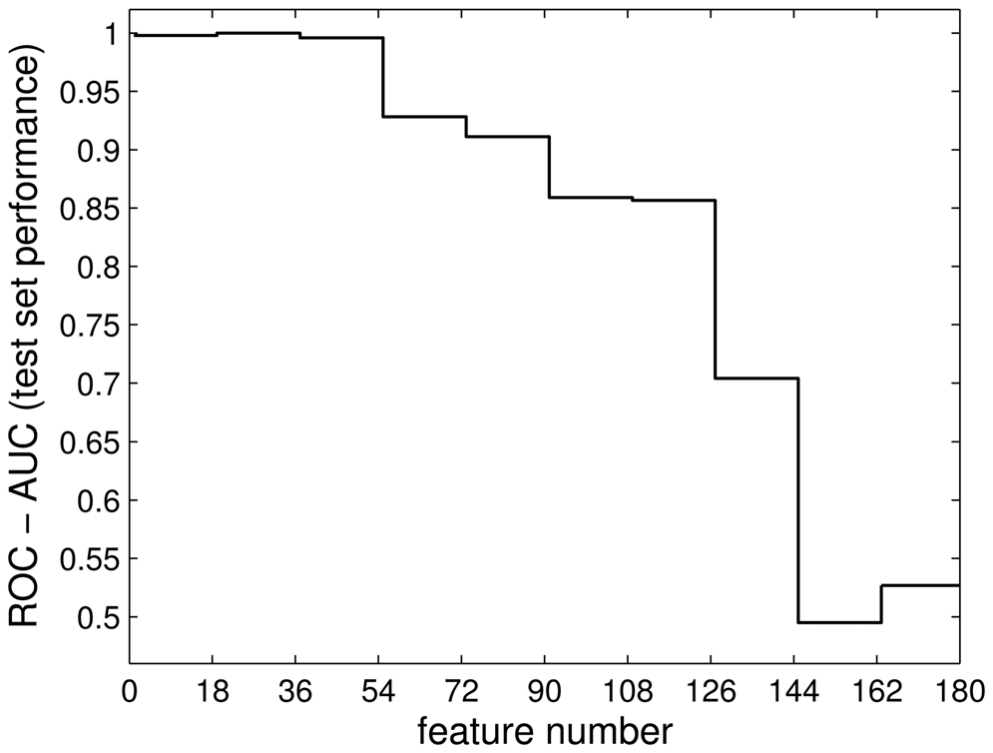
Test set performance (AUC of ROC) for the GMLVQ system restricted to subsequent subsets of 18 features which are ordered according to diagonal relevance 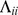. The AUC deteriorates from values close to 1 for highly relevant features to AUC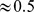 when using 18 features of low relevance.

Obviously, the outcome and interpretation of relevance parameters depends on the precise form of the distance measure, Eq. (3), or more generally, on the parameterization of the classifier. For instance, systems with diagonal matrix 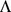 could only take into account single features and would disregard the discriminative power of particular pairs of features. Accordingly, featurs which display low relevance in our scheme might become significant in more complex classifiers. Nevertheless we believe that our method provides valuable insight into the discriminative power of features and pairs of features. The following simple experiment further illustrates this claim: We ranked features according to the corresponding 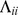 and restricted the obtained GMLVQ classifier to the use of only 18 features for classification. All other features were omitted when evaluating distances and scores, cf. Eqs. (3,5), no re-training of the system was performed. The restricted classifier was evaluated in terms of its test set ROC. Close to perfect test set classification with an AUC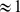 was retained when using only the leading 18 features which all are derived from the above mentioned 7 markers. It is interesting to note that also the following two subsets of 18 features, i.e. with relevance ranks 19–36 and 37–54, yielded excellent test set performance. Figure 9 shows how the resulting AUC decreases for subsequent subsets of 18 features with decreasing relevance. Performance deteriorated when subsets of features with very low relevance were used, resulting in essentially random class assignments with AUC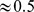.

A more reliable determination of discriminative markers, and even more so, the selection of a minimal set of features for correct classification would require systematic validation studies including the re-training of the GMLVQ system on the respective feature sets. Due to the limitations of the data provided in the challenge we postponed this line of research to forthcoming studies.

### Outlook

More challenging data sets will have to be inspected to further demonstrate the usefulness of the approach in the analysis of flow cytometry data. This should, of course, include the systematic comparison with other methods. A comparison of various classifiers in the context of the FlowCAP2/DREAM6 challenge can be found in [1].

The identification of leukemia subtypes in a larger study population requires the introduction of several prototypes representing the class of AML patients. The extension of GMLVQ in terms of localized distance measures [9], [11] appears also promising in this context.

The reliable identification of feature relevances for marker selection should also be based on larger, more representative data sets. For a successful application of GMLVQ for bio-marker selection in the context of tumor classification see [27]. The application of multi-class, potentially localized, GMLVQ will open new routes to the identification of discriminative markers in the differential diagnosis of AML subtypes. In forthcoming studies, the consideration of histogram specific distance measures will also be studied along the lines of [15].

The analysis presented here was based on the entire cell population of a given subject. More general problems, including the above mentioned identification of AML subtypes, might require an analysis on the level of individual cells. We intend to consider the development of prototype based automated gating procedures in forthcoming projects.

### Available Software

The specific Matlab code used to generate our contribution to the DREAM6/FlowCAP2 *Molecular Classification of Acute Myeloid Leukemia Challenge 2011* is publicly available at http://www.the-dream-project.org/story/code [3].

A Matlab toolbox *Relevance and Matrix adaptation in Learning Vector Quantization*, including GMLVQ and important variants, is made available at http://matlabserver.cs.rug.nl/gmlvqweb/web/ [32].
